# Cluster of Falciparum Malaria Cases in UK Airport

**DOI:** 10.3201/eid1408.080031

**Published:** 2008-08

**Authors:** Alison J. Rodger, Graham S. Cooke, Rosalynn Ord, Colin J. Sutherland, Geoffrey Pasvol

**Affiliations:** * Northwick Park Hospital, Harrow, UK; † University College Long, London, UK; ‡ Africa Centre for Health and Population Studies, Somkhele, South Africa; § Imperial College London, London, UK; ¶ Hospital for Tropical Diseases, London, UK

**Keywords:** malaria, Plasmodium falciparum, molecular genotyping, outbreak, dispatch

## Abstract

A cluster of 6 cases of *Plasmodium falciparum* malaria occurred in a UK airport among 30 travelers returning to the United States from East Africa. Molecular genotyping analysis indicated that all had been exposed to different parasites. The travelers’ use of chemoprophylaxis was poor; their perception of risk was limited.

Although most malaria occurs in Africa and Asia, it is also found in travelers returning from malaria-endemic regions (*1*). In the United States in 2005, a total of 1,528 cases of malaria were diagnosed, an increase of 15% from the previous year (*2*). In the United Kingdom in 2006, a total of 1,758 malaria cases were reported (*3*). In each country, malaria prophylaxis was inadequate for 75%–80% of patients (*2*,*3*).

Most cases of malaria in travelers occur sporadically as single cases. We describe a cluster of *Plasmodium falciparum* malaria cases among a group of US citizens returning from East Africa.

## The Cases

A group of 30 US citizens spent June and July 2005 in Kenya and Uganda as part of an educational program. Their return journey took them from Nairobi to London, where they spent a week before their flight to the United States. By the time they arrived at the airport departure lounge, 6 persons had become sick with high fevers, rigors, and some confusion. They were referred by airport staff to the Infectious Diseases Unit at Northwick Park Hospital, London.

All arrived at the unit within 3 hours of referral and were proven to have falciparum malaria on peripheral blood smears. Clinical features of persons with disease are shown in the Table. Of these 6 persons, all were 19–22 years of age, and 5 exhibited initial clinical features of severe malaria, e.g., impaired consciousness (disorientation as to time, place, or person; or prostration [inability to sit up and drink]). These 5 persons required intravenous quinine and intensive monitoring; the other person had less severe disease and was treated with a combination of oral atovaquone and proguanil. All improved by day 4 and returned to the United States shortly thereafter.

**Table:** Clinical data for 6 US travelers with falciparum malaria returning from East Africa, 2005*

Patient no.	Malaria severity (*4*)	Temp, °C	BP, mm Hg	Parasitemia, %	HGB†	Platelets‡	Creatinine§	Bilirubin¶	ALT#	Base excess**
1	Severe	38.2	97/52	3.5	9.3	33	89	71	103	–2.4
2	Severe	40.1	132/73	3.5	16.0	38	108	40	101	3.1
3	Mild	38.0	110/75	1.0	13.3	48	70	20	34	ND
4	Severe	36.4	99/63	3.5	16.5	193	111	22	34	–2.3
5	Severe	39.4	105/72	1.5	16.8	121	103	20	38	-0.8
6	Severe	37.8	79/53	1.0	13.1	54	102	9	41	–4.8

*Date of symptom onset for each patient was 2005 Jul 20. Temp, body temperature; BP, blood pressure; HGB, hemoglobin; ALT, alanine aminotransferase; ND, not done. †Reference range 11–15 g/dL. ‡Reference range 120–308 × 10^9^/L. §Reference range <100 μm/L. To convert to mg/dL, divide by 88.4. ¶Reference range <19 μm/L. To convert to mg/dL, divide by 17.1. #Reference range 19–50 U/L. **In mmol/L. To convert to mEq/L, divide by 1.0.

In light of these cases, all other members of the group who had returned to the United States were alerted to contact their healthcare providers if they became symptomatic. Another person became febrile while in the United States and was initially treated with conventional antimicrobial drugs; this person was later recalled to a hospital and treated appropriately for falciparum malaria after the diagnosis of his colleagues became known. Unconfirmed reports were received that 2 more patients had received treatment for malaria (species unidentified) within 6 months of return. The high attack rate in this group of students and the close temporal clustering of cases led us to examine the possibility that the group had experienced a mini-outbreak as a result of a point-source exposure.

A study-specific, self-administered questionnaire gathered information about demographics, onset and nature of symptoms, medical history, malaria protective measures, and chemoprophylaxis and adherence. The questionnaire was given to patients 1–6 in the UK hospital and to patient 7 by email. Clinical data for patients 1–6 were obtained during their UK hospital stay, and clinical data for patient 7 was obtained from the diagnosing and treating physician in the United States. Genetic diversity of parasite isolates was investigated by PCR amplification of polymorphic regions of the *P. falciparum* merozoite surface protein 1 (*Pfmsp1*) and *Pfmsp2* loci as previously described (*5*).

The group had traveled together through Kenya and Uganda, with almost identical itineraries. All had slept in shared accommodations under unimpregnated malaria nets. Despite weekly malaria education sessions, antimalarial drug use was not widespread in the absence of a clear predeparture recommendation. Only 1 of 7 patients had taken malaria prophylaxis, but this person did not continue using chemoprophylaxis for the full period at risk. None of the 7 patients recalled being bitten by mosquitoes. However, when returning to Nairobi on July 2, 2005, the group was delayed for several hours by the roadside in the rice-growing area of Mwea (northern Kenya). Many had little protective clothing with them and while waiting until dusk that evening, noticed a large number of mosquitoes of unknown species around them. This event occurred 20 days before first onset of symptoms. After visiting Uganda, the group returned to Nairobi on July 11, arrived in London on July 13, and went to London’s Heathrow Airport on July 20 for the flight back to the United States.

Paired peripheral blood samples from pretreatment and day 1 posttreatment were provided by 6 of the 7 patients; for the other patient, only a pretreatment sample was available for DNA analysis. No 2 patients had identical alleles at both *Pfmsp1* and *Pfmsp2* loci (Figure). At least 12 different parasite genotypes were detected*.* Pretreatment and posttreatment samples were similar except for those of patient 2, for whom postquinine allelic patterns differed for *Pfmsp1* and *Pfmsp2* on day 1 and day 0.

**Figure:**
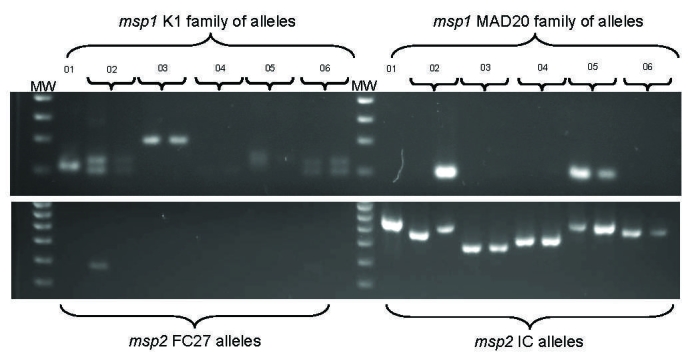
Molecular typing of malarial parasites from 6 US travelers with falciparum malaria returning from East Africa in 2005. *Plasmodium falciparum* merozoite surface protein 1 (*Pfmsp1*) (upper gel) and *Pfmsp2* (lower gel) allelic variation among isolates was determined by nested PCR and agarose gel electrophoresis. DNA markers (MW) are in lanes 1 and 13 in each gel. Two family-specific primer sets were used for each of the 2 genes. No parasites of the Ro33 allelic family of *msp1* were found (data not shown). Pretreatment (day 0) isolates are shown for all patients. Posttreatment (day 1) parasite isolates are also shown for patients 2–6.

## Conclusions

Malaria-endemic areas, such as East Africa, are increasingly popular destinations for US travelers. Therefore, suboptimal adherence to malaria chemoprophylaxis and effective preventative measures against mosquito bites (such as use of bed nets) is cause for concern (*3*). Antimalarial drug use was poor among the case-patients, for whom emphasis was placed on personal protective measures rather than chemoprophylaxis. The high attack rate for this group is consistent with rates reported by other studies demonstrating the injudicious risk of traveling to a malaria-endemic area without taking effective antimalarial drugs (*6*,*7*).

The course of disease for patients 1–6 displayed remarkable synchrony; onset of symptoms occurred within 24 hours of each other and illness proceeded rapidly. These 6 patients received prompt diagnosis and treatment at a specialist unit and recovered fully. Had it had not been for alert airport staff, these 6 patients (5 with severe malaria) would have been on a 9-hour flight to the United States; consequences for the patients could have been fatal. These cases highlight the benefits of vigilant staff at airports, even for passengers boarding from non–disease-endemic areas. Prompt contact tracing enabled a missed diagnosis of malaria to be rectified at a US facility and highlights the advantage of actively seeking all those who might have shared the same exposure.

The clustering of the onset of symptoms for patients 1–6 suggested a common timing of infection, although other unproven factors, such as the effect of atmospheric pressure changes during air travel, may have led to the synchronous onset of the cases. Molecular evidence suggests that despite synchrony of symptoms, the genetically heterogeneous parasite variants were unlikely to have come from 1 infectious mosquito. We estimate that at least 12 distinct haploid parasite genotypes were circulating among the 6 patients. Our data also demonstrate, for patient 2, that circulating parasite genotypes can change profoundly immediately after treatment, as previously observed for quinine-treated persons (*8*). Caution is therefore required when interpreting genotyping data for treated persons (*9*).

The only reported intense exposure to mosquitoes was during the travel delay in Mwea. The main rice-growing season starts in June, which is also the time of *Anopheles* spp. peak abundance (*10*). However, the mosquitoes seen at Mwea were not positively identified as *Anopheles*, and although the students did not recall being bitten by mosquitoes on other occasions, we cannot rule out infection later in the journey, which would be more probable given the date of onset of symptoms in nonimmune persons.

This mini-outbreak demonstrates the need to encourage travelers to take malaria prophylaxis and to improve their knowledge about the risk of malaria in their area of proposed travel. It also underscores the value of alerting fellow travelers in the same party to the possibility of contracting malaria.
